# Clinical decision-making and care pathways for people with multiple long-term conditions admitted to hospital: a scoping review

**DOI:** 10.1136/bmjopen-2025-100270

**Published:** 2025-08-04

**Authors:** Nicola L Howe, Ella-Rose Blackburn, Amelia Sheppard, Sara Pretorius, Jana Suklan, Sue Bellass, Rachel Cooper, Suzy Gallier, Elizabeth Sapey, Avan A P Sayer, Miles Witham

**Affiliations:** 1 NIHR HealthTech Research Centre in Diagnostic and Technology Evaluation, Newcastle University, Newcastle upon Tyne, UK; 2 AGE Research Group, Translational and Clinical Research Institute, Newcastle University, Newcastle upon Tyne, UK; 3 NIHR Newcastle Biomedical Research Centre, Newcastle Upon Tyne Hospitals NHS Foundation Trust, Newcastle upon Tyne, UK; 4 PIONEER Hub, University of Birmingham, Birmingham, UK; 5 Institute of Inflammation and Ageing, University of Birmingham, Birmingham, UK

**Keywords:** Multimorbidity, Clinical Decision-Making, Clinical Reasoning

## Abstract

**Abstract:**

**Objectives:**

People living with multiple long-term conditions (MLTC) admitted to hospital have worse outcomes and report lower satisfaction with care. Understanding how people living with MLTC admitted to the hospital are cared for is a key step in redesigning systems to better meet their needs. This scoping review aimed to identify existing evidence regarding clinical decision-making and care pathways for people with MLTC admitted to the hospital. In addition, we described research methods used to investigate hospital care for people living with MLTC.

**Design:**

A scoping review methodological framework formed the basis of this review. We took a narrative approach to describe our study findings.

**Data sources:**

A search of Medline, Embase and PsycInfo electronic databases in July 2024 captured relevant literature published from 1996 to 2024.

**Eligibility criteria:**

Studies that explored care pathways and clinical decision-making for people living with MLTC or co-morbidities, studies conducted fully or primarily in secondary or tertiary care published in English Language and with full text available.

**Data extraction and synthesis:**

Titles and abstracts were independently screened by two authors. Extracted data included country of origin, aims, study design, any use of an analytical framework or design, type of analyses performed, setting, participant group, number of participants included, health condition(s) studied and main findings. Included studies were categorised as either: studies reviewing existing literature, studies reviewing guidance, studies utilising qualitative methods or ‘other’.

**Results:**

A total of 521 articles were screened, 17 of which met the inclusion criteria. We identified a range of investigative methods. Eight studies used qualitative methods (interviews or focus groups), four were guideline reviews, four were literature reviews and one was classified as ‘other’. Often, researchers choose to combine methods, gathering evidence both empirically and from reviews of existing evidence or guidelines. However, none of the empirical qualitative studies directly or solely investigated clinical decision-making when treating people living with MLTC in acute care and the emergency department. Studies identified complexities in care for people living with MLTC, and some authors attempted to make their own recommendations or draft their own guidance to counter these.

**Conclusions:**

This scoping review highlights the limitations of the current evidence base, which, while diverse in methods, provides sparse insights into clinical decision-making and care pathways for people living with MLTC admitted to hospital. Further research is recommended, including reviews of guidelines and gathering insights from both healthcare professionals and people living with MLTC.

**STRENGTHS AND LIMITATIONS OF THIS STUDY**

- Identification of a notable research gap; that is, the investigation of how clinicians make real-time decisions for individuals with multiple long-term conditions.
- The broad focus applied, which was inclusive of different study methodologies, enabled us to understand the landscape of research and evidence synthesis in this topic area.
- Exclusion of literature from primary care and community settings which was not congruent with our research programme but could have provided additional relevant evidence for hospital care.

## Background

 Multiple long-term conditions (MLTC), also known as multimorbidity, are defined as the presence of two or more chronic health conditions.^1^ MLTC are common; globally, a third of adults are estimated to have MLTC^2^ and their prevalence is projected to increase further,^3^ with the proportion of those aged 65 and over in England with MLTC increasing from 54% in 2015 to an estimated 68% by 2035.^2^

The challenges posed by MLTC, including the need for ongoing treatments and assessments, influence both physical and emotional health, as well as overall quality of life and social engagement. Furthermore, MLTC place significant demand on health and social care services with higher rates of emergency department (ED) presentation and unplanned hospital admission.^4^,^7^ People living with MLTC who are admitted to hospital are also more likely than people with no or only a single LTC to experience extended stays, clinical complications, premature mortality, reliance on polypharmacy, adverse drug reactions, problems of care co-ordination and reduced quality of life.^1 3 8 9^ They also report lower satisfaction with care.^10^

MLTC thus represents a challenge to healthcare systems, which, especially in hospitals, are largely optimised to deal with single health conditions.^11^ To improve how hospitals and health services can provide care for people living with MLTC admitted to hospital, it is important to understand how MLTC are currently managed within hospitals, how staff make clinical decisions regarding referrals and care pathways for people living with MLTC arriving in the ED, and how these decisions are impacted by the specific care challenges that MLTC present. There is also a need to understand how these pathways could be improved to facilitate the care of people living with MLTC and what currently works well, so that these aspects of the hospital care system can be maximised accordingly. The ADMISSION Research Collaborative^12^ was set up specifically to research MLTC in people admitted to hospital, and this scoping review forms part of a programme of work seeking to understand pathways of care within hospitals and how these differ for people living with MLTC.

This scoping review aimed to identify and summarise literature which has explored clinical decision-making and care pathways for people with MLTC admitted to hospital, and to identify the methodologies previously used to research this.

## Methods

The scoping review was carried out following a protocol shared between project partners (unpublished) based on the scoping review methodological framework outlined by Arksey and O’Malley^13^ and updated guidance on scoping review methodology.^14^ This scoping review was reported according to the Preferred Reporting Items for Systematic Reviews and Meta-Analyses extension for Scoping Reviews (PRISMA-ScR).^15^

### Identifying the research question

The main purpose of this review was to identify and summarise published research on clinical care pathways and clinical decision-making for people with MLTC admitted to hospital. In particular, we wanted to identify work that had been conducted previously to examine how staff working in hospital care (also referred to as secondary care) make clinical decisions on admissions, referrals and care for people with MLTC. The secondary aim of this review was to identify methodological approaches (including study design and analytical frameworks) taken to investigate MLTC. The review question was therefore *‘what research evidence exists regarding care, care pathways Or clinical decision-making for people with MLTC in secondary care and what methods have been used to investigate these?’*

### Identifying relevant studies

The search strategy for the review was developed by E-RB and JS and was reviewed by SP. The review aimed to locate published studies defined by the inclusion criteria. Three databases accessed via Ovid (MEDLINE, EMBASE and PsycInfo) were selected for their relevance to medical literature and searched systematically. Papers already known to researchers or identified serendipitously and thought to be potentially eligible were imported from Google Scholar (n=27). Initial pilot searches of the databases identified articles relevant to the review question and enabled refinement of the search strings for each database to ensure the capture of relevant literature. The search terms were selected to identify studies focusing on populations with multiple long-term or coexisting conditions, examining their pathways through healthcare services (such as care pathways, patient journeys or referrals) and exploring aspects of clinical decision-making. This approach aimed to capture research on how healthcare professionals (HCPs) make decisions for these patients within care pathways.

The final search terms used were: (comorbid* or co-morbid* or multimorbid* or multi-morbid* or multiple long or coexist* or co-exist*) AND (care pathway* or patient pathway* or referral*) OR (decision-making or clinical decision-making). Specific search strings for Medline, Embase and PsycInfo are available in online supplemental tables 1–3 respectively. The search strategy, including all identified keywords and index terms, was adapted for each database. The search was conducted in August 2024 using Medline 1996 to July week 4 2024, Medline 1996 to week 31 and PsycInfo 2002 to July week 4 2024. We limited the search to records from 1996 onwards to increase the likelihood of identifying the terms ‘multimorbidity’ and ‘multiple long-term conditions’, terms which came into use only relatively recently. Our initial pilot searches indicated that these phrases were much less likely to appear in literature published prior to 1996. The search strategy and inclusion criteria contained no specifications regarding named combinations of specific diagnoses or co-morbidities, though we did include such studies if they met the inclusion criteria.

Inclusion criteria were studies that explored care pathways and clinical decision-making for people living with MLTC or co-morbidities, studies conducted fully or primarily in secondary or tertiary care, published in English Language and with full text available. Studies were excluded if they did not focus on aspects of healthcare or clinical decision-making (‘wrong outcomes’ in PRISMA), were conducted in settings *fully* outside of secondary care (eg, primary care, community care), were not published in English and studies where the full text was not available. Protocols, conference abstracts, posters, comment and opinion publications were also excluded.

### Study selection

Once the searches had been completed, the identified article titles and abstracts were uploaded to Covidence^16^ where results were deduplicated and independently screened against the inclusion and exclusion criteria before data were extracted. One of the 17 included articles was identified during hand searching as the identified conference abstract did not have an associated full text. No further author or citation searches were made.

The initial screening was completed by E-RB and AS with JS acting as a second screener. Disagreements about inclusion were addressed via discussions between JS, NLH and SP or involved arbitration with another team member.

### Data extraction

Relevant summary data from the publications that met the inclusion criteria were extracted into Covidence.^16^ This involved taking the information of interest from each article into a custom data extraction form. The data extracted from relevant papers included: lead author, year, country of origin, aims, study design, any use of an analytical framework or design, type of analyses performed, setting, participant group, number of participants included, health condition(s) studied and the study’s main findings. Data extraction was carried out by E-RB and AS and was checked by second reviewers, SP and NLH. No critical appraisal of data sources, or analysis of data was conducted as per established scoping review methodology.^14^

### Charting the data

The extracted data were then separated into four categories:

1. Studies reviewing existing literature.
2. Studies reviewing guidance.
3. Studies using qualitative methods such as focus groups or interviews.
4. All other studies.

**Figure 1:**
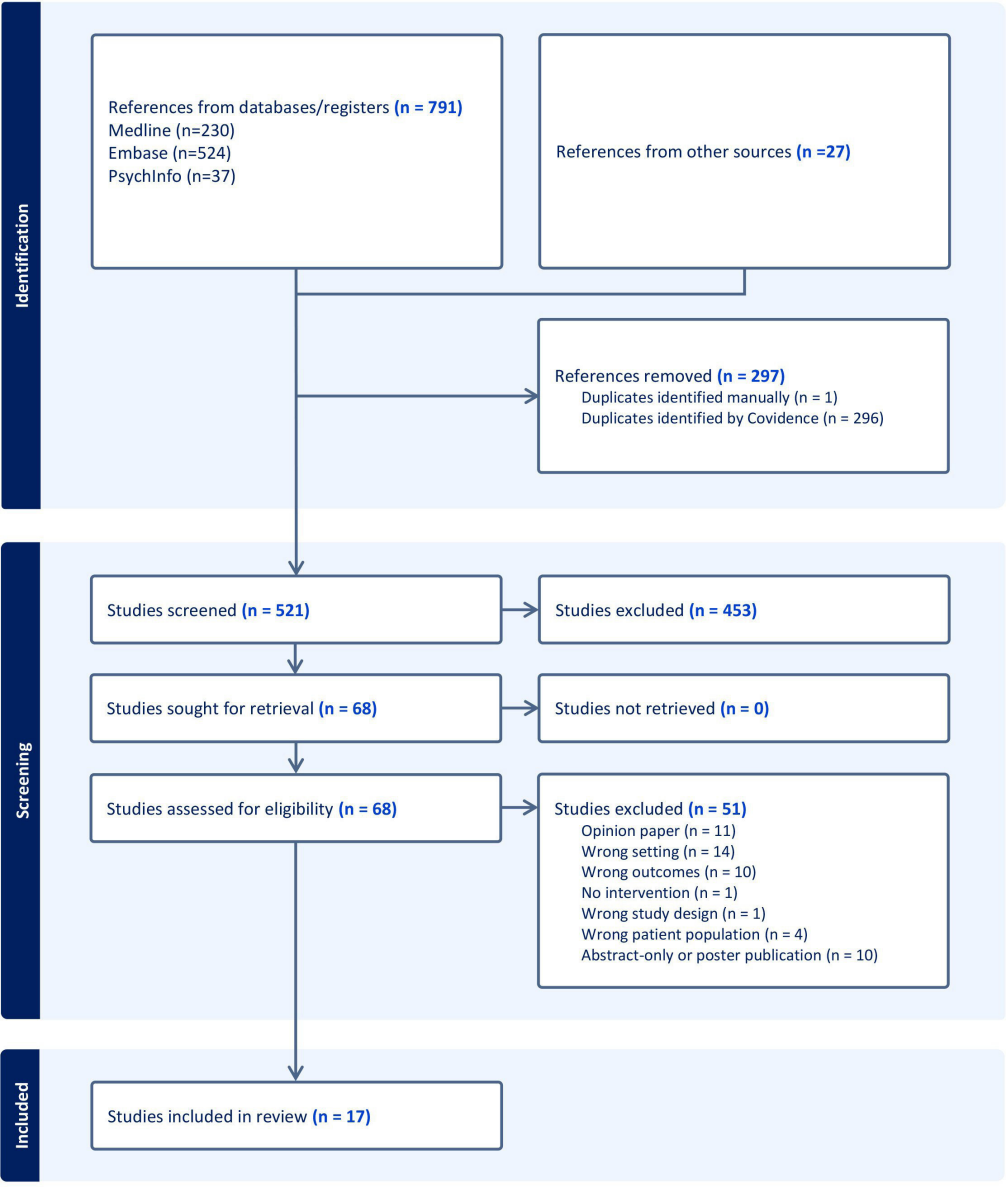
Preferred Reporting Items for Systematic Reviews and Meta-Analyses flow diagram.

The reasons for exclusion and associated counts are noted in figure 1.

### Collation, summarisation and reporting of results

Aligned with guidance on methodological approach to scoping reviews,^13^ the data from included studies have not undergone thematic analysis but are presented as a descriptive, narrative summary of included studies, separated into four distinct categories as described above.

### Patient and public involvement

The ADMISSION Research Collaborative benefits from the input of a patient advisory group (PAG), including people living with MLTC and carers. The PAG was involved from the inception of the study and again at the end of the research. At the start, the group helped us refine our research question by highlighting the relevance of hospital care and ensuring it addressed issues important to patients, particularly the complexity of navigating hospital care. At the end, we met again to present the results of the research, interpretation of findings and the identification of research gaps and to understand best practices for dissemination of the research findings to a wider audience. The authors would like to thank the PAG for their invaluable feedback that has helped to shape the focus of the work, of which this review forms a part.

## Results

### Overview of studies

After deduplication, a total of 521 articles were identified and screened against the predefined inclusion and exclusion criteria. A total of 17 articles^17^,^33^ met the inclusion criteria, with 51 records being excluded in accordance with the exclusion criteria after full text review. Included studies comprised eight original qualitative studies,^17^,^24^ four guideline reviews,^2326^,^28^ four literature reviews^29^,^3133^ and one ‘other’ study type; a policy brief drawn from gathering evidence from existing care programmes.^32^

Included studies spanned eight individual countries, all of which were high income, with the largest proportion of studies being from the UK (n=5), followed by Australia (n=3) and the USA (n=2).

The majority of papers (n=9) took a general approach to MLTC and were not diagnosis-specific.^2125 27^,^33^ One study focused on pregnancy and comorbidities.^18^ The remaining studies focused on two or more specified conditions (eg, psychosis and diabetes).^1719 20 22^,^24 26^ Participant groups in the qualitative studies consisted of either purely HCPs,^1719^,^21 25^ or a combination of HCPs and patients.^18 22 24^ Only one study included informal caregivers as participants.^22^

The studies that included patient participant groups typically collected data on the participants’ experiences of healthcare related to their MLTC within varied settings including secondary care,^18 19^ both primary and secondary (hospital) care,^25 30 33^ and tertiary care^20 22^ or a combination of all three.^21^ Although studies (21) touched on decision-making in secondary care for patients with MLTC, none of the empirical qualitative studies directly or solely investigated clinical decision-making when treating people living with MLTC in acute care and the emergency department. Extracted data from each study are displayed in online supplemental table 4.

### Studies reviewing existing literature

The included literature reviews^29^,^3133^ had various structures, consisting of a systematic review,^33^ a scoping review,^31^ a ‘review of the implications of multimorbidity for the design of health system’^29^ and a ‘scoping study’ which combined a systematic literature search with a qualitative thematic analysis of the identified literature.^30^ A range of conceptual and analytical approaches was used across the included studies. One study developed conceptual models of comorbidity and multimorbidity, examining their implications for patients and health systems, and how these models might inform system design.^29^ Another identified and categorised interventions for people with multimorbidity or for delivering patient-centred care in primary care, resulting in a taxonomy of interventions.^31^ A further systematic review provided a narrative synthesis of evidence on multimorbidity, spanning definitions to interventions.^33^ Doessing and Burau applied De Jonge *et al*’s model of complexity to thematically organise their findings, distinguishing between *case complexity* (patient characteristics) and *care complexity* (challenges in care delivery).^30^

The reviews demonstrated the complexity and challenges of care for people living with MLTC, including heterogeneity in definitions of MLTC, limited guidance for HCPs in how to approach and make decisions for people with MLTC, gaps in understanding how to provide patient-centred care, especially with regard to involving patients in clinical decision-making and lack of evidence from low- and middle-income countries (LMICs), which limits the global applicability and equity of the evidence base.

In primary and secondary care, coordinated, collaborative, patient-oriented approaches, self-management support for patients and the development of training for HCPs have the greatest potential to result in positive impacts for people living with MLTC.^29^,^31^ Consideration and inclusion of people living with MLTC and their families in the design of and decisions about their own care is key.^29 33^ One study highlighted the need for further prospective research (eg, longitudinal cohort studies or randomised control trials) to provide more definitive evidence on individuals with MLTC which will inform both clinical practice and policy making.^33^

### Studies reviewing guidance

All four of the included guideline reviews^2326^,^28^ went beyond an initial review of guidance and incorporated additional analyses. Two reviews used expert opinions either through a ‘workshop-based consensus meeting’^27^ or an expert panel to provide feedback on issues identified in the review.^28^ Hughes *et al* selected just five NICE guidelines and then followed them for two hypothetical patients to illustrate the potential cumulative impact of applying single disease recommendations to people with multimorbidity.^26^ The most in-depth review examined guidelines and data relating to three conditions and performed economic modelling.^23^

Authors suggested that the existing guidelines for treating people living with MLTC are limited in their application to MLTC, rarely allowing for MLTC and are based on inadequate clinical evidence.^23 26 28^ For example, clinical trials which provide evidence informing guidelines are often based on populations which exclude those who have comorbidities or are older (and therefore more likely to have comorbidities).^23^ Strictly following selected clinical guidelines for hypothetical patients was demonstrated to lead to adverse outcomes.^26^ Ultimately, the included guideline reviews point towards the need for HCPs to adapt and refine guidelines, adopting individualised care as the best way to manage people living with complex MLTC.^27 28^

### Studies using qualitative methods such as focus groups or interviews

Nearly half of the papers included (n=8) used qualitative methodologies,^17^,^2224 25^ with six studies using semi-structured interviews,^17^,^1921 22 25^ one study using focus groups^24^ and one using a combination of both.^20^ These studies each had a generic qualitative design, using some form of thematic analysis,^17^,^2022 25^ content analysis^19 24^ or a constant-comparison approach.^20 21^ With regard to qualitative analytical frameworks, models and approaches, one study developed its interview guide based on established principles for managing multimorbidity in older adults, as outlined by the American Geriatrics Society such as incorporating patient preferences, acknowledging gaps in evidence, weighing treatment risks and benefits, evaluating feasibility and aiming to optimise outcomes and quality of life for older adults.^21^ One study used a grounded theory approach to analyse interview data from a vignette-based study on deviations from clinical guidelines in MLTC.^17^ Researchers identified, compared and refined themes to develop a theoretical model of clinical decision-making.^17^ Another study employed interpretivist thematic analysis, using theoretical lenses to explore how clinicians make sense of their decisions within social and organisational contexts.^18^ The interview-based studies focused predominantly on quality of care, coordination of care, care pathways, and the relevance, or lack thereof, of guidelines to treating MLTC. Five of the eight qualitative studies explored the views of HCPs and three studies^18 22 24^ incorporated views of patients as well as those of HCPs.

The included qualitative evidence provided research into all sectors of care for people living with MLTC, with two studies set in secondary care,^18 19^ two in primary and secondary care in conjunction,^24 25^ and two in tertiary care.^20 22^ One paper investigated a combination of primary, secondary and tertiary care.^21^ The remaining paper described a study where the setting was unclear but involved a sample of 100 physiotherapists who are known to work in a variety of settings including secondary care, primary care and community settings.^17^

### Clinical decision-making for patients with MLTC in secondary care

We aimed to explore existing research on how healthcare staff in hospital settings make clinical decisions about admissions, referrals and ongoing care for people living with MLTC. However, we found that the literature did not explicitly focus on decision-making processes themselves. Instead, it largely explored related themes that provide indirect insights into the context, influences and structures that shape decision-making in practice.

Much of the literature underscored the complexity of managing MLTC, which stems from the interaction of multiple conditions, polypharmacy and the absence of clear, applicable clinical guidelines.^2326^,^28 30 33^ Clinical decisions are often made in a context where existing evidence and care models are oriented toward single diseases, limiting their relevance for people with multimorbidity.^23 26 33^ Both HCPs and patients acknowledged the importance of managing conditions effectively and providing holistic care for individuals with MLTC, recognising also that MLTC leads to challenges in treatment and care coordination.^19^,^21^ Guidelines and current care pathways often failed to account for comorbidities. As a result, HCPs are often required to depart from standard protocols and develop individualised care plans based on clinical judgement and patient need.^17^ Care for people living with MLTC was seen as lacking as compared with care for patients with single conditions and the literature indicated a possible lack of overarching accountability or responsibility for people living with MLTC.^21^ The studies also highlighted structural barriers such as poor continuity and coordination of care.^21^ HCPs reported feeling that increased training for staff could help them to adapt care for people living with MLTC.^19^ Multidisciplinary and integrated models of care were frequently proposed as solutions,^18 20 22^ with an emphasis on communication, collaboration^20 30^ and valuing both professional expertise and patient input. Yet, such models were not widely implemented or evaluated and professionals often lacked the support systems, such as decision aids or access to specialist input, that would enable more effective, patient-centred decision-making.^22 25 30 32 33^

Finally, the literature reflected a persistent gap in patient involvement in decision-making, despite growing recognition of the importance of shared goals and collaborative care.^33^ Efforts to support patient-centred care, including goal-setting and self-management interventions, were unevenly adopted and insufficiently tailored to the realities of multimorbidity.^25 31^ Together, these findings suggest that while clinicians are aware of the challenges and the need for personalised, collaborative approaches, they operate within systems and structures that constrain their ability to consistently deliver such care.

As per the literature reviews, there was recognition that a preventative and collaborative approach which included patients in decisions about their own care was essential for improving decision-making, care quality and patient experience for individuals with comorbidities or MLTC.^18^,^2022 25^ Other potential strategies could include appointing a dedicated ‘care coordinator’,^18 21^ considering individual patients’ needs,^22^ supporting self-management and preventative care,^20^ integrating specific services^19^ and educating patients about how their conditions interact with one another.^19^

### Other studies

The other included study was a policy brief drawn from gathering quantitative evidence from existing care programmes.^32^ Similar to other included studies, a patient-centric or individualised approach was recommended for people living with MLTC. Implications for guidelines and policy-makers as well as for HCPs in delivering personalised care tailored to the needs of these patients.^32^ To overcome these challenges in care, the study highlighted strategies such as providing education and professional training, enacting policy and legislative changes to establish new professional roles focused on care coordination, improving information exchange, revising privacy protection laws to facilitate patient information sharing across organisations and securing funding to support the development of integrated care.^32^

## Discussion

This scoping review found only a limited body of work relevant to the topic of decision-making and care pathways for people with MLTC admitted to the hospital. Existing studies were based in high-income countries, and only a minority of studies focused on the patients themselves. More work had been conducted on guidelines than in other areas we examined, with very little work on clinical decision-making when people living with MLTC are admitted to the hospital.

Our scoping review included two previous scoping reviews.^30 31^ Both were conducted several years ago and new studies have been published since. Although both reviews had some overlap with the current review, their focus differed. Doessing and Burau^30^ focused on care coordination, conducting a scoping review of literature with a qualitative synthesis. Their key conclusion was that complexity was the hallmark of care for people with MLTC, which needed to be either reduced or embraced if care was to meet the needs of people living with MLTC. Poitras *et al*^31^ focused on elements of care interventions that might be effective in improving health outcomes for people living with MLTC. They reported a taxonomy of elements and although this included elements on care management and decision-making processes, this was not the primary focus of the review, nor did the review seek to encompass wider literature describing pathways and processes of care in the hospital setting.

Our review revealed that although few studies directly examined how hospital staff make decisions for people with MLTC, related literature highlights key contextual and structural influences. Overall, decision-making for people with MLTC takes place in a context of clinical uncertainty, constrained guidance and underdeveloped support structures, underscoring the need for more integrated, flexible and patient-centred approaches.

A recurring theme was the significant challenge of caring for patients with MLTC. Studies described difficulties in delivering patient-centred care within the constraints of standardised systems, managing the clinical complexity of multimorbidity, ensuring coordination and continuity across different services and the occasional reluctance among professionals to take ownership of patients with complex needs. These challenges often intersect with concerns about maintaining quality of care for this population, highlighting systemic pressures and the need for more tailored approaches.

Another prominent finding was the need for flexibility in clinical practice. HCPs often found it necessary to adapt care beyond the bounds of standard guidelines. While guidelines play an important role in supporting decision-making, included studies pointed out that these are frequently designed around single-disease models and are therefore not well suited to individuals with multiple conditions. In some cases, the underlying research used to develop clinical recommendations excluded patients with MLTC, leading to limited applicability in real-world, complex cases. As a result, staff described instances where they had to bend or deviate from existing protocols, drawing on their clinical judgement and experience to deliver appropriate care.

Finally, the literature frequently referred to ideal models of care that implicitly influence or support decision-making. These included shared decision-making approaches that emphasise patient priorities and personalised care planning, as well as collaborative models such as multidisciplinary or integrated care. Strong communication pathways, preventative approaches and support for patient self-management were also highlighted as foundational to effective care. Although these models do not provide direct insight into moment-to-moment decision-making, they offer valuable perspectives on the organisational and philosophical frameworks that shape how decisions are approached and enacted. Importantly, these challenges are not exclusive to healthcare for individuals with MLTC. Similar issues are evident across other areas of care, particularly for people living with chronic illnesses who often also experience comorbidities. For example, research on diabetes management has emphasised the need to shift away from a narrow focus on glycaemic control within a single specialist setting, towards more interdisciplinary care that reflects the condition’s complexity and the associated risks of cardiovascular complications and comorbidities.^34^ Published literature has also emphasised the importance of personalised, patient-centred care for individuals with long-term conditions more broadly.^35^,^37^ These parallels suggest that the fragmentation of care, limitations of disease-specific guidelines and the need for flexible, person-centred decision-making are widespread issues in chronic disease management more broadly.

Key strengths of our study were the broad focus that we applied which was inclusive of different study methodologies, enabling us to understand the broad landscape of research and evidence synthesis in this topic area. A number of limitations also need to be acknowledged. We chose to exclude literature solely from primary care and community settings as this was not the focus of enquiry for our research programme. More MLTC research has been done in primary care settings and information from this setting is still likely to be at least partly relevant to the hospital setting. We did not attempt an exhaustive search of specific combinations of conditions, as our main intention was to examine MLTC or comorbidity as a broader entity. This decision was taken both for practical reasons (the number of combinations to search for would make the task unmanageable) but also for philosophical reasons; specific combinations of conditions can be viewed as an extension of the current paradigm of managing each single condition one at a time rather than engaging with alternative, more holistic approaches that the presence of MLTC may require. Searching for and including information from studies examining specific combinations of conditions could nevertheless still inform decision-making around people living with MLTC who are admitted to the hospital.

It is clear from our review findings that there is both a need and an opportunity for more research in this area. There is a notable gap in research that explicitly explores how clinicians make real-time decisions for individuals with MLTC. A potential direction for future study could involve ethnographic research, observing clinicians within their natural working environments to gain insight into decision-making as it unfolds. Alternatively, vignette-based studies where clinicians are asked to describe their decision-making processes in response to realistic patient scenarios could offer valuable, practice-based perspectives that closely reflect real-time clinical reasoning. Quantitative studies describing care pathways in the hospital for people living with MLTC, qualitative studies with both patients and clinicians to explore the decision-making process around care and care pathways for people with MLTC, and a focus on designing better guidelines that take into account MLTC, are all areas requiring further research. Such research should not be confined to high-income countries; the burden of MLTC in LMICs is already considerable^5^ and likely to increase in the next decade and secondary healthcare systems in these countries also need to prepare to change to meet the needs of people living with MLTC.

## Supplementary material

10.1136/bmjopen-2025-100270 online supplemental file 1

## Data Availability

Data are available upon reasonable request. All data relevant to the study are included in the article or uploaded as supplementary information.
