# Supplementary material for: Clinical decision-making and care pathways for people with multiple long-term conditions admitted to hospital: a scoping review
Source: BMJ Open. 2025 Aug 4;15(8):e100270. doi: 10.1136/bmjopen-2025-100270 (PMC12323535; doi:10.1136/bmjopen-2025-100270)
Supplement: online supplemental file 1 [file bmjopen-15-8-s001.docx]

**Supplementary Material**

**Supplementary Table 1**

| Ovid Medline <1996 to July Week 4 2024> | | |
| --- | --- | --- |
|  | **Search Term** | **Results** |
| 1 | ((comorbid* or co-morbid* or multimorbid* or multi-morbid* or **co-exist*** or **coexist*** or multiple long) adj2 (long-term or long-lasting or chronic)).mp. [mp=title, book title, abstract, original title, name of substance word, subject heading word, floating sub-heading word, keyword heading word, organism supplementary concept word, protocol supplementary concept word, rare disease supplementary concept word, unique identifier, synonyms, population supplementary concept word, anatomy supplementary concept word] | 5388 |
| 2 | (care pathway* or patient pathway* or referral*).mp. [mp=title, book title, abstract, original title, name of substance word, subject heading word, floating sub-heading word, keyword heading word, organism supplementary concept word, protocol supplementary concept word, rare disease supplementary concept word, unique identifier, synonyms, population supplementary concept word, anatomy supplementary concept word] | 158679 |
| 3 | (decision making or clinical decision making).mp. [mp=title, book title, abstract, original title, name of substance word, subject heading word, floating sub-heading word, keyword heading word, organism supplementary concept word, protocol supplementary concept word, rare disease supplementary concept word, unique identifier, synonyms, population supplementary concept word, anatomy supplementary concept word] | 228879 |
| 4 | 2 or 3 | 381610 |
| 5 | 1 and 4 | 230 |

**Supplementary Table 2**

| Ovid Embase <1996 to 2024 Week 31 | | |
| --- | --- | --- |
|  | **Search Term** | **Results** |
| 1 | ((comorbid* or co-morbid* or multimorbid* or multi-morbid* or multiple long or **co-exist*** or **coexist***) adj2 (long-term or long-lasting or chronic)).mp. [mp=title, abstract, heading word, drug trade name, original title, device manufacturer, drug manufacturer, device trade name, keyword heading word, floating subheading word, candidate term word] | 10583 |
| 2 | (care pathway* or patient pathway* or referral*).mp. [mp=title, abstract, heading word, drug trade name, original title, device manufacturer, drug manufacturer, device trade name, keyword heading word, floating subheading word, candidate term word] | 325447 |
| 3 | (decision making or clinical decision making).mp. [mp=title, abstract, heading word, drug trade name, original title, device manufacturer, drug manufacturer, device trade name, keyword heading word, floating subheading word, candidate term word] | 565839 |
| 4 | 2 or 3 | 876829 |
| 5 | 1 and 4 | 524 |

**Supplementary Table 3**

| Ovid APA PsycInfo <2002 to July Week 5 2024> | | |
| --- | --- | --- |
|  | **Search Term** | **Results** |
| 1 | ((comorbid* or co-morbid* or multimorbid* or multi-morbid* or **co-exist*** or **coexist*** multiple long) adj2 (long-term or long-lasting or chronic)).mp. [mp=title, abstract, heading word, table of contents, key concepts, original title, tests & measures, mesh word] | 1059 |
| 2 | (care pathway* or patient pathway* or referral*).mp. [mp=title, abstract, heading word, table of contents, key concepts, original title, tests & measures, mesh word] | 31309 |
| 3 | (decision making or clinical decision making).mp. [mp=title, abstract, heading word, table of contents, key concepts, original title, tests & measures, mesh word] | 127502 |
| 4 | 2 or 3 | 157154 |
| 5 | 1 and 4 | 37 |

**Supplementary Table 4**

| **Lead author, year, title and reference number** | **Country** | **Aim(s)** | **Study design** | **Analytical framework/ design** | **Methodology and data analysis** | **Setting** | **Study Participant group** | **Number of participants** | **Health condition(s)** | **Main findings** |
| --- | --- | --- | --- | --- | --- | --- | --- | --- | --- | --- |
| Boyd, 2010, Future of multimorbidity research: How should understanding of multimorbidity inform health system design? [29] | United States of America | To review the implications of multimorbidity for the design of health system and to understand the research needs for this population. | Review article | Metrics of applicability to individuals aged 65 years or older with multiple comorbid diseases | Review | N/A | N/A | N/A | No specific diagnosis | People with multimorbidity should receive a patient-centred approach to care throughout the health system. Understanding  how to deliver this type of care in effective and efficient ways is an enormous challenge, and opportunity, for clinicians, researchers, and policy makers today. |
| Doessing, 2015, Care coordination of multimorbidity: a scoping study [30] | Denmark | The study addresses two issues: the characteristics and preconditions of care coordination for patients with  multimorbidity; and the factors that promote or inhibit care coordination at the levels of provider organizations and healthcare professional | Scoping study | Jonge et al's model of complexity (case and care complexity) | Combines a systematic  literature search with a qualitative thematic analysis. | Primary and secondary care | N/A | N/A | No specific diagnosis | Multimorbidity is a key challenge for healthcare systems worldwide, and there is a large number of patients who  suffer from multimorbidity. As this scoping study demonstrates, this challenge revolves around complexity. The importance of collaboration and communication among healthcare professionals is high. |
| Dorenkamp, 2016, Comorbidities force physiotherapists to deviate from guidelines: A vignette study [17] | The Netherlands | To examine whether physiotherapists adapt recommended treatment where comorbidity is present | Mixed methods | Inductive thematic analysis based on grounded theory | Interviews guided by vignettes  Quantified % of PTs who changed practice  Thematic analysis | Not specified | Physical therapists (PTs) | 100 claudication network members | Intermittent claudication (in isolation (vignette 1), with comorbid COPD (vignette 2), with comorbid COPD and knee osteoarthritis (vignette 3) | Thirty percent of PTs did not adjust treatment despite comorbidity, 30% adapted treatment plan in light of comorbidity. Comorbidity induced 40% of PTs to abandon guideline recommendations and create individualised treatment plan based on the health needs of the patient as described by the vignette. |
| Guthrie 2017, Better guidelines for better care: accounting for multimorbidity in clinical guidelines “a structured examination of exemplar guidelines and health economic modelling” [23] | United Kingdom | To test the feasibility of new approaches to developing single-disease guidelines to better account for multimorbidity. | Literature-based and economic modelling project. | Three examplar questions re accounting for comorbidity | Guideline analysis focused on the National Institute for Health and Care Excellence (NICE) and the Scottish Intercollegiate Guidelines Network (SIGN).  Systematically collated and summarised evidence /evaluated exploratory modelling methods. | N/A | N/A | N/A | Type 2 diabetes, depression and heart failure | Comorbidity was rarely accounted for in the clinical research questions that framed the development of the exemplar guidelines and was rarely accounted for in treatment recommendations. For all three conditions, the trials underpinning treatment recommendations largely excluded older, more comorbid and more co-prescribed patients. |
| Hanley, 2024, Lost in the System: Responsibilisation and Burden for Women With Multiple Long-Term Health Conditions During Pregnancy. [18] | United Kingdom | To explore experiences and (health)care of women with MLTC during pregnancy. | Qualitative | Interpretivist thematic analysis | Semi-structured interviews  Thematic analysis | Secondary care | Women who were at least 28 weeks pregnant or had given birth in the last 2 years. Healthcare professionals with experience caring for pregnant women. | 57 women, 51 healthcare professionals. | MLTC and pregnancy | Findings illustrate the importance of multidisciplinary care, where clinicians acknowledge their professional limits, women's expertise and identities are valued, and they are treated as partners in their care. |
| Hughes, 2013, Guidelines for people not for diseases: the challenges of applying UK clinical guidelines to people with multimorbidity [26] | United Kingdom | To examine the extent to which National Institute of Health and Clinical Excellence (NICE) guidelines address patient comorbidity, patient centred care and patient compliance to treatment recommendations. | Guideline analysis | Semi-quantitative (score for extent to which guidelines accounted for people with comorbidities) | Guideline review followed by application of guidelines to two hypothetical patients with multimorbidity. | N/A | N/A | N/A | Type-2 diabetes mellitus, secondary prevention for people with myocardial infarction, osteoarthritis, chronic obstructive pulmonary disease and depression. | Comorbidity was inconsistently accounted for in the guidelines. Clinical guidelines are limited in their focus on single diseases and the evidence upon which guideline recommendations are based upon apply only to subsets of the population. Explicitly following clinical guidelines for two hypothetical patients with physical and mental health comorbidities produced complex treatment regimes with a significant risk of adverse drug reactions. |
| Hultsjö, 2013, Mental healthcare staff's knowledge and experiences of diabetes care for persons with psychosis “a qualitative interview study” [19] | Sweden | To explore mental healthcare staff's experiences of diabetes care given to people with psychosis. | Qualitative | Inductive thematic analysis (not mapped to specific framework) | Semi-structured interviews  Qualitative content analysis | Secondary care (psychiatric outpatients) | HCPs | 12 | Psychosis and diabetes | Staff were aware of the risks of type 2 diabetes among their patients and therefore performed lifestyle interventions to promote these. Staff felt there was a lack of training among diabetes nurses to adapt diabetes care to suit persons with cognitive dysfunctions. |
| Lo, 2016, Primary and tertiary health professionals views on the health-care of patients with co-morbid diabetes and chronic kidney disease “a qualitative study”[20] | Australia | To explore the perspectives of general practitioners and tertiary health-care professionals concerning key factors influencing healthcare of diabetes and Chronic Kidney Disease (CKD). | Qualitative | Generic inductive thematic approach (thematic analysis) | Cross-sectional Interviews & focus groups  Thematic analysis | Four tertiary health services in two Australian cities | HCPs | 65 | Diabetes and CKD | An integrated specialist diabetes-kidney service could improve care. Supporting GPs with an accessible, multidisciplinary diabetes-renal health service underpinned by strong communication pathways, a preventive approach and quality improvement activities, may improve health-care and patient outcomes in co-morbid diabetes and CKD. |
| McNamara, 2017, Health professional perspectives on the management of multimorbidity and polypharmacy for older patients in Australia [21] | Australia | To explore current approaches to multimorbidity management, and perceived barriers and enablers to deliver appropriate medications management for community-dwelling patients with multimorbidity and polypharmacy, from a broad range of healthcare professional (HCP) perspectives in Australia. | Qualitative | "American Geriatrics Society principles for  multimorbidity" | Semi-structured interviews  Analysed using a constant comparison approach. | HCPs working in metropolitan and rural areas of Victoria and South Australia (primary, secondary and tertiary care). | HCPs | 26 | No specific diagnosis | Participants perceived multimorbidity management as important. Challenges with coordination and continuity of care, pressures of workload and poorly defined individual responsibilities for care, all contributed to participants avoiding ownership of multimorbidity management. |
| McWilliams, 2018, Cancer-related information needs and treatment decision-making experiences of people with dementia in England: a multiple perspective qualitative study [22] | United Kingdom | To explore cancer-related information needs and decision-making experiences of patients with cancer and co-morbid dementia, their caregivers and oncology HCPs | Cross-sectional qualitative | Thematic analysis framework (inductive) presented as cancer pathway trajectory | Semi-structured Interviews informed by topic guide.  Thematic analysis. | Regional tertiary care cancer centre | Patients, HCPs, Informal caregivers | 31 (10 patients, 9 informal caregivers, 12 HCPs) | Cancer and dementia | Appropriate models of care are required. Oncology teams should strive to involve healthcare staff with dementia expertise as early as possible in the cancer pathway. |
| Muth, 2019, Evidence supporting the best clinical management of patients with multimorbidity and polypharmacy: a systematic guideline review and expert consensus [27] | United Kingdom | To identify and analyse available evidence-based clinical practice guidelines for multimorbidity or polypharmacy in order to investigate the clinical decision support they provide and the key concepts they address. | Systematic guideline review followed by expert consensus exercise | Pre-defined framework based on Ariadne principles - ideal framework | Systematic search for existing clinical practice guidelines.  Thematic analysis conducted on guidelines and recommendations extracted. | N/A | N/A | N/A | No specific diagnosis | Eight guidelines were included (four each on multimorbidity and polypharmacy) 250 recommendations were extracted. Decisions about health care for patients with multimorbidity require a more individualized approach that considers outcomes across conditions. |
| Poitras, 2018, What are the effective elements in patient-centered and multimorbidity care? A scoping review [31] | Canada | To identify the specific elements of patient-centered care and multimorbidity interventions that are associated with positive outcomes for patients. | Scoping review | Taxonomy of interventions | Inductive analysis | N/A | N/A | N/A | No specific diagnosis | Patient-oriented approaches, self-management support interventions and developing training for HCP's were the most frequent interventions with a potential to result in positive impacts for patients with chronic conditions. These conditions must address the concerns of multimorbidity. |
| Rijken, 2017, How to improve care for people with multimorbidity in Europe? [32] | Germany | To improve care for people with multiple chronic conditions (multimorbidity) in European countries. | Policy brief drawn from a mix of methodologies | Framework visualizing patient-centred integrated care for people with multimorbidity | Information was gathered on 101 innovative care programs in 24 European countries. | N/A | N/A | N/A | No specific diagnosis | Research on the implementation and effectiveness of patient-centered integrated care for people with multimorbidity is scarce. Care organizations could implement decision support systems that highlight appropriate treatment options for individual patients. |
| Schiotz, 2017, Quality of care for people with multimorbidity “a case series” [24] | Denmark | To investigate quality of care for people with multimorbidity in the publicly funded healthcare system in Denmark. | Case series with planned chart review | Questionnaire assessing care (HC services provided and medical treatment) | Medical record review & Focus groups.  Qualitative content analysis. | Primary care sector, the hospital sector, and the municipality of Copenhagen for individuals with multimorbidity. | Patients with multimorbidity   HCP's | 23 patient records included in full-record review  9 HCPs | Cardiovascular disease, unstable angina, stable angina, heart failure, type 1 diabetes, COPD, depression | The care provided to approximately two-thirds of the patients did not take comorbidities into account and insufficiently addressed more diffuse symptoms or problems. Findings reveal quality of care deficiencies for people with multimorbidity. |
| Uhlig, 2014, A framework for crafting clinical practice guidelines that are relevant to the care and management of people with multimorbidity [28] | United States of America | To present consensus- based recommendations for guideline developers to make guidelines more useful for the care of people with multimorbidity. | Review article | Iterative process informed by review of key literature and experience. | Guideline development steps - review, expert consensus and modified Delphi  of key literature and experience. | N/A | N/A | N/A | No specific diagnosis. | Many of the recommended steps depend on ability of a CPG workgroup to make complex judgments, often in the absence of high-quality evidence. For HCP's for patients with multimorbidity, these recommendations will require validation through implementation, evaluation and refinement. |
| Vermunt, 2018, A three-goal model for patients with multimorbidity: A qualitative approach [25] | The Netherlands | To develop conceptual descriptions of goal-oriented care  by examining the perspectives of general practitioners (GPs) and clinical geriatricians  (CGs), and how the concept relates to collaborative communication and shared decision-making with elderly patients with multimorbidity. | Qualitative | Inductive thematic analysis (not mapped to specific framework) | Semi-structured interviews guided by topic guide.  Thematic analysis. | Primary and secondary care | GPs and CGs | 15 GPs  18 CGs | No specific diagnosis | Reasons to explicate goals are the need-to-know individual patient values in case of multimorbidity, including management in acute  situations. The proposed model could facilitate collaborative goal-setting for patients with multiple long-term conditions in clinical practice. |
| Xu, 2017, Evidence on multimorbidity from definition to intervention: An overview of systematic reviews [33] | Australia | To better understand the existing evidence on multimorbidity. | Systematic review | Pre-defined categories: definitions, measurement, prevalence, risk factors, health outcomes, clinical practice, medication and intervention and management | Systematic literature search  Data extraction and review. | Evidence collected from studies in various settings (primary, secondary). | N/A | 53 studies included for review. | No specific diagnosis. | Patients are often excluded in clinical decision-making. Most guidelines did not take into account patients with multimorbidity.  Interventions on multimorbidity were varied. |
