# Supplementary material for: Clinical decision-making and care pathways for people with multiple long-term conditions admitted to hospital: a scoping review
Source: BMJ Open. 2025 Aug 4;15(8):e100270. doi: 10.1136/bmjopen-2025-100270 (PMC12323535; doi:10.1136/bmjopen-2025-100270)
Supplement: Supplementary file 2 [file bmjopen-2025-100270.reviewer_comments.pdf]

PEER REVIEW HISTORY

BMJ Open publishes all reviews undertaken for accepted manuscripts. Reviewers are asked to complete a checklist review form and are provided with free text boxes to elaborate on their assessment. These free text comments are reproduced below.

ARTICLE DETAILS

Title (Provisional)

Clinical decision-making and care pathways for people with multiple long-term conditions admitted to hospital: a scoping review

Authors

Howe, Nicola L; Blackburn, Ella-Rose; Sheppard, Amelia; Pretorius, Sara; Suklan, Jana; Bellass, Sue; Cooper, Rachel; Gallier, Suzy; Sapey, Elizabeth; Sayer, Avan AP; Witham, Miles

---

VERSION 1 - REVIEW

**Reviewer**	**1**
**Name**	**Zippel-Schultz, Bettina**
**Affiliation**	**The German Foundation for the Chronically Ill**
**Date**	**25-Feb-2025**
**COI**	**None**

The authors address the important questions of what care pathways exist for multimorbid patients, particularly in hospitals, whether these pathways are appropriate, how decisions are made within them, and how understanding these processes can improve the care of multimorbid patients. To analyze these questions, a scoping review was conducted. The study aimed, firstly, to identify previous research findings related to care pathways and clinical decision-making for multimorbid patients and, secondly, to describe the methods used.

I have some questions and comments:

Generally, the term clinical decision making could be misleading in this context, as it includes diagnosis, severity assessment, and treatment management. However, in this article, it only refers to admission, referrals, and care. This discrepancy might have caused the search to focus on different topics than intended. Especially since it was a mandatory term in the search strategy.

Search and search strategy:

• Why was the search limited to the years 1996–2024? A rationale for this selection would be helpful.

• Use of MeSH Terms: Why were MeSH terms not used in the search strategy?

• The terms clinical decision making and decision making should be enclosed in quotation marks to ensure they are recognized as related terms in Medline.

• If “or” was written in lowercase, it may not have been recognized as a logical operator, potentially leading to incorrect search results.

• The description of the search strategy in the text (lines 20–24) does not match supplementary table 1 in the appendix, where the link was not made exclusively via AND.

• Please add the number of references per database at the top of the flowchart for better transparency.

Abstract, results and discussion:

• The abstract primarily reports methodological findings, while from my perspective the study results could be described in more detail.

• The discussion could be expanded. Topics such as stronger communication and collaboration between stakeholders, patient-centered care approaches, or gaps in knowledge (as are mentioned in table 1) are also discussed in other areas of the healthcare system (e.g., in the management of chronically ill patients, who are often also multimorbid). These parallels could be explored further.

• The research gap identified in the discussion closely resembles the initial research question. It may be useful to highlight a particularly urgent or interesting aspect that warrants further investigation in future studies. It is true that studies should not only be conducted in high-income countries, but there are also more concrete challenges in these countries that should be addressed, e.g. as mentioned above.

Overall, the article provides an important analysis of a relevant topic but contains some methodological ambiguities and aspects that should be clarified or elaborated.

**Reviewer**	**2**
**Name**	**Eubank, Breda**
**Affiliation**	**Mount Royal University, Health & Physical Education**
**Date**	**20-Mar-2025**
**COI**	**None**

Thank you for the opportunity to review this scoping reivew on the clinical decision-making and care pathways for people with MLTC. This is an interesting topic. Overall, the manuscript is well-written, succint, and clear.

I have two suggestions.

First, your scoping review proposes to answer 2 questions. 1) To understand what work had been conducted previously to examine how staff working in

hospital care (also referred to as secondary care) make clinical decisions on admissions, referrals and care for people with MLTC; and 2) To identify methodological approaches (including study design and any analytical frameworks) taken to investigating MLTC. The manuscript does a good job of speaking to your secondary question. However, in your results and discussion, I do not see any content relating to your primary question. Either you need to adjust your research question to only include your secondary question, or add data and content to answer your primary question.

Second, I do not see a critical appraisal framework in your methods or the results or an evidence appraisal. This could entail a grading of the evidence exercise and/or risk of bias assessment.

VERSION 1 - AUTHOR RESPONSE

**Comments to the Author:**<br>The authors address the important questions of what care pathways exist for multimorbid patients, particularly in hospitals, whether these pathways are appropriate, how decisions are made within them, and how understanding these processes can improve the care of multimorbid patients. To analyze these questions, a scoping review was conducted. The study aimed, firstly, to identify previous research findings related to care pathways and clinical decision-making for multimorbid patients and, secondly, to describe the methods used.	We would like to thank the reviewer for taking the time to review our manuscript. We have made amendments where applicable and respond to each of your comments below.
Generally, the term clinical decision making could be misleading in this context, as it includes diagnosis, severity assessment, and treatment management. However, in this article, it only refers to admission, referrals, and care. This discrepancy might have caused the search to focus on different topics than intended. Especially since it was a mandatory term in the search strategy.	Initial piloting and exploration of search terms allowed us to optimise our search terms as far as possible. We needed to include the term 'decision making' in order to identify studies concerning decisions in MLTC care. We were particularly interested in identifying studies exploring decision making in referrals and admissions in secondary care but we do accept that decision making also refers to decisions around diagnosis, severity assessment and treatment management and would have been happy to review studies exploring these aspects of MLTC care. However, we identified few

	studies overall that explored MLTC decision making in any context.
**Search and search strategy:**<br>Why was the search limited to the years 1996–2024? A rationale for this selection would be helpful.	1996 to present date (or 2002 for PsychINFO) was chosen to search the databases via Ovid. We rejected use of 1946 or 1988 to present date as from our experience (and piloting) we would be much less likely to identify terms such as MLTC or multimorbidity in the literature earlier than 1996. We have amended the methods to explain this (page 7, paragraph 2).
Use of MeSH Terms: Why were MeSH terms not used in the search strategy?	When developing the search strategy, we consulted with a specialist librarian. We decided to rely on free-text search terms to maximise sensitivity and inclusiveness of the scoping review. Our aim was to capture a heterogeneous body of literature on clinical decision-making and care pathways for people with multiple long-term conditions (MLTC), including studies that may not yet be indexed with appropriate MeSH terms due to indexing lag, inconsistent indexing or discrepancies in interdisciplinary terminology. There are also several limitations of doing so, such as reduced precision, missing synonyms or variations of the terms or lack of hierarchical searching.
The terms clinical decision making and decision making should be enclosed in quotation marks to ensure they are recognized as related terms in Medline.	We have re-run the searches and can confirm that quotation marks do not affect the retrieval, since MEDLINE via Ovid does not treat quotation marks as phrase search indicators. Instead, it automatically interprets multiple adjacent words as a phrase when appropriate.
If "or" was written in lowercase, it may not have been recognized as a logical operator, potentially leading to incorrect search results.	As above we have re-run the search and can confirm that the lower case 'or' did not prevent it being seen as a logical operator.
The description of the search strategy in the text (lines 20–24) does not match supplementary table 1 in the appendix, where the link was not made exclusively via AND.	We have amended the description of the search terms in the manuscript to match the supplementary tables (page 7, paragraph 2).
Please add the number of references per database at the top of the flowchart for better transparency.	We have edited the PRISMA diagram to include the number of records from each source for transparency. Whilst doing this we noted that we had submitted the search terms/strategy from 2023 instead of our latest search in 2024 and have updated this in the supplementary materials.
**Abstract, results and discussion:**<br>The abstract primarily reports methodological findings, while from my	We agree and have amended the abstract so that the results section focuses more on the results of the included studies and less on the methodological approach or findings.

perspective the study results could be described in more detail.	
The discussion could be expanded. Topics such as stronger communication and collaboration between stakeholders, patient-centered care approaches, or gaps in knowledge (as are mentioned in table 1) are also discussed in other areas of the healthcare system (e.g., in the management of chronically ill patients, who are often also multimorbid). These parallels could be explored further.	We would like to thank the reviewer for their helpful suggestion to strengthen the contextual framing of our discussion. We have incorporated this into the revised discussion (page 22-23, paragraph 2 onwards).
The research gap identified in the discussion closely resembles the initial research question.<br>It may be useful to highlight a particularly urgent or interesting aspect that warrants further investigation in future studies. It is true that studies should not only be conducted in high-income countries, but there are also more concrete challenges in these countries that should be addressed, e.g. as mentioned above.	It was intentional that the identified research gap echoed the research question. As above, we have added some text to the discussion to emphasise that we were not able to fully answer our first aim, (to examine how staff working in hospital care make clinical decisions on admissions, referrals and care for people with MLTC). We hope this leads more clearly to our suggestion that the key research gap is in fact for more qualitative studies examining this (page 21, paragraph 3 onwards).
Overall, the article provides an important analysis of a relevant topic but contains some methodological ambiguities and aspects that should be clarified or elaborated.	Thank you, we hope that we have now addressed these satisfactorily and that the methodology is more transparent.
Thank you for the opportunity to review this scoping reivew on the clinical decision-making and care pathways for people with MLTC. This is an interesting topic. Overall, the manuscript is well-written, succint, and clear.	We would like to thank the reviewer for taking the time to review our manuscript. We have made amendments where applicable and respond to your comments below.
First, your scoping review proposes to answer 2 questions. 1) To understand what work had been conducted previously to examine how staff working in hospital care (also referred to as secondary care) make clinical decisions on admissions, referrals and care for people with MLTC; and 2) To identify methodological approaches (including study design and any analytical frameworks) taken to investigating MLTC.<br>The manuscript does a good job of speaking to your secondary question. However, in your results and discussion, I do not see any content relating to your primary question. Either you need to adjust your research question to only include your secondary	As stated in our results section, we found no clear evidence addressing research question 1 (what research has been conducted on clinical decision making for admissions or referral of patients with MLTC from the perspective of secondary care staff). We did identify that some of the evidence discusses subjects which impact upon decision making processes.<br>We have amended the text in the discussion to make it clearer that none of the included studies directly addressed research question 1.<br><br>However, we agree that we could expand upon this in the discussion, and we have added text to make it more explicit that we did not find very much evidence addressing research question 1 (page 21, paragraph 3 onwards).

question, or add data and content to answer your primary question.	Later in the discussion we do address the need for further research into clinical decision making.
Second, I do not see a critical appraisal framework in your methods or the results or an evidence appraisal. This could entail a grading of the evidence exercise and/or risk of bias assessment.	This was a scoping review, conducted using widely-accepted methods established by Arksey and O'Malley (https://doi.org/10.1080/1364557032000119616), in which critical appraisal (including bias assessment) of the included evidence is not considered appropriate. The purpose of our scoping review is to present the evidence identified. We also believe that the range of studies included is too broad and varied for the results of such an exercise to be meaningful. We have however conducted our scoping review in accordance with PRISMA-ScR to ensure thorough reporting.

VERSION 2 - REVIEW

**Reviewer**	**2**
**Name**	**Eubank, Breda**
**Affiliation**	**Mount Royal University, Health & Physical Education**
**Date**	**20-May-2025**
**COI**	

Thank you for the opportunity to review this synthesis of findings from a scoping review exploring pathways and clinical decision making for people living with multiple long-term conditions.

I think this is an important topic, but the reseach question needs to be more specific. The question is presented as "aiming to summarize the existing evidence regarding clinical decision making and care pathways for MLTC". As I began reading, I had interpreted this to mean a descriptive synthesis of the different pathways and frameworks for MTLC. However, as I read through the results, you present on the efficacy and effectiveness of pathways for MTLC. There is minimal synthesis on your actual primary purpose which is to "understand what work has been conducted previously to examine how staff working in hospital care make clinical decisions on admissions, referrals, and care".

- In some areas, you use "decision-making". In others you use "decision making". Please choose one.

- When reporting the country of origin of your included studies (n=17), your math only adds up to 10: 5+3+2.

- Can you please clarify what you mean by "wrong outcome"? The preceding sentence (line 52) is clear as you are not including studies that "focused on aspects of health or clinical decision-making", but I do not understand what the connection to "wrong outcomes" is. Especially as you later report on health and adverse outcomes.

- In your results section (page 11, paragraph 2), please add in reference numbers to indicate which papers were: general; which focused on 2 or more specified conditions; which were qualitative of purely HC professionals; and which were a combo of HC professionals and patients.

- Studies conducted in secondary and tertiary care centres were part of your exclusion criteria, yet you included references 18, 19, 20 and 22.

- There are several discussion and concluding remarks embedded within your results section. Under each of the sections (existing literature, guidance, qualitative). The first paragraphs of each section summarized the evidence from the literature, but the subsequent paragraphs provided discussive or general remarks. For instance, page 11, last paragraph reads " the reviews demostrate complexity and challenges of care for people living with MLTC". Perhaps you can present the specific complexities and challenges instead.

- In your discussion, you mention that "most used qualtiative methods" with inference that this was a negative point. Why is this the case? Qualitative studies are rich with patient and provider experiences and perspectives that cannot otherwise be elucidated from quantiative methods. Your manuscript would be richer if you synethesized the analytic frameworks of your studies in your results (as this was your secondary aim) and discussed the significance of qualitative work.

- Again, the biggest limitation to this manuscript is the lack of synthesis and information provided that actually describes "how healthcare staff in hospital settings make clinical decisions about admissions, referrals, and ongoing care". To me this would require a desription of the actual pathways or frameworks guiding the decision-making process. Your Table 4 is quite robust and provides some of this insight. Perhaps you might want to translate some of that information into your results section.

VERSION 2 - AUTHOR RESPONSE

Comment	Response to reviewer
Thank you for the opportunity to review this synthesis of findings from a scoping review exploring pathways and clinical decision making for people living with multiple long-term conditions.	Thank you for taking the time to review our manuscript, we appreciate your suggestions, have responded to your comments and made meaningful changes to the manuscript.

I think this is an important topic, but the research question needs to be more specific. The question is presented as "aiming to summarize the existing evidence regarding clinical decision making and care pathways for MLTC". As I began reading, I had interpreted this to mean a descriptive synthesis of the different pathways and frameworks for MTLC. However, as I read through the results, you present on the efficacy and effectiveness of pathways for MTLC. There is minimal synthesis on your actual primary purpose which is to "understand what work has been conducted previously to examine how staff working in hospital care make clinical decisions on admissions, referrals, and care".	Thank you for pointing this out. We agree that we could have been clearer about our aim. As a scoping review, our aim was to not to synthesise but to describe the work that has been conducted in this field so far. We have amended our manuscript to remove the word synthesis and amended where applicable the description of the aims in the abstract and the methods sections to better reflect this.
- In some areas, you use "decision-making". In others you use "decision making". Please choose one.	Thank you for spotting this. We have amended the manuscript to read "decision-making" in all instances.
- When reporting the country of origin of your included studies (n=17), your math only adds up to 10: 5+3+2.	We have reported countries with the highest number of studies and have restructured the sentence to reflect this more clearly. Full results on country of origin are available in table of results (supplementary table 4).
- Can you please clarify what you mean by "wrong outcome"? The preceding sentence (line 52) is clear as you are not including studies that "focused on aspects of health or clinical decision-making", but I do not understand what the connection to "wrong outcomes" is. Especially as you later report on health and adverse outcomes.	Thank you for your comment and for highlighting the confusion around the phrase "wrong outcomes." We agree that the wording in this sentence was unclear and may have caused misunderstanding. To clarify, studies that **did not** focus on aspects of health care or clinical decision-making were excluded because their primary outcomes were not relevant to our research question. We have now revised the sentence to more clearly reflect this criterion. "Wrong outcomes" is how this is expressed in the PRISMA diagram. We have amended the text in the manuscript to read ""(wrong outcomes" in PRISMA diagram)" to make this clearer. The other reasons for exclusion in the PRISMA diagram more clearly and obviously map to the reasons for exclusion listed in the manuscript.
- In your results section (page 11, paragraph 2), please add in reference numbers to indicate which papers were: general; which focused on 2 or more specified conditions; which were qualitative of purely HC professionals; and which were a combo of HC professionals and patients.	We have added in the reference numbers to the results paragraph 2 under the heading 'overview of studies'.<br><br>We have also added the numbers of qualitative studies which utilised the views of HCPs and which also included the views of patients in the results under the section 'Studies utilising

	qualitative methods such as focus groups or interviews'.
- Studies conducted in secondary and tertiary care centres were part of your exclusion criteria, yet you included references 18, 19, 20 and 22.	Studies conducted in secondary care were one of our inclusion criteria. We excluded studies conducted exclusively or solely in primary or community care centres. We included studies which were conducted in a mixture of settings as long as one of those settings included secondary care, which is the case in the mentioned studies.
- There are several discussion and concluding remarks embedded within your results section. Under each of the sections (existing literature, guidance, qualitative). The first paragraphs of each section summarized the evidence from the literature, but the subsequent paragraphs provided discussive or general remarks. For instance, page 11, last paragraph reads " the reviews demostrate complexity and challenges of care for people living with MLTC". Perhaps you can present the specific complexities and challenges instead.	We have tried to summarise the identified evidence (under each section), giving an overview of key or majority findings from each type of methodological approach, giving examples as appropriate. The sentence highlighted by the reviewer concludes with some examples of the challenges and complexities of care for people with MLTC and we have extended this to account for all of the challenges identified within the literature reviews included.
- In your discussion, you mention that "most used qualtiative methods" with inference that this was a negative point. Why is this the case? Qualitative studies are rich with patient and provider experiences and perspectives that cannot otherwise be elucidated from quantiative methods. Your manuscript would be richer if you synethesized the analytic frameworks of your studies in your results (as this was your secondary aim) and discussed the significance of qualitative work.	We agree that the use of qualitative methods by the majority of studies is not a negative, and in fact is the most appropriate way of investigating care pathways and decision-making for care of patients with MLTC. We have removed the phrase "used mostly qualitative methods".<br><br>Thank you for the suggestion about the synthesis of the analytic frameworks as a secondary aim. In line with this suggestion, we have expanded the results section within the 'Studies reviewing existing literature' and 'Studies utilising qualitative methods such as focus groups or interviews' headings giving the reader some additional descriptive detail on the models and analytical frameworks used.
- Again, the biggest limitation to this manuscript is the lack of synthesis and information provided that actually describes "how healthcare staff in hospital settings make clinical decisions about admissions, referrals, and ongoing care". To me this would require a desription of the actual pathways or frameworks guiding the decision-making process.	The results section has been expanded to include a summary of the findings from the included studies. As previously highlighted in the discussion section, despite focusing our search on research investigating clinical decision-making in hospital care, the majority of identified literature did not focus on decision-making but instead on the context, influences and structures that shape decision-making. The additional text describes the findings around these influencing factors.
Your Table 4 is quite robust and provides some of this insight. Perhaps you might want	We thank you for your comment on Table 4. We have added additional detail to the results section in order to outline the frameworks, models and

to translate some of that information into your results section.	analytical approaches used in the identified papers and to describe some of the key findings from these studies.

VERSION 3 - REVIEW

Reviewer 2

Name Eubank, Breda

Affiliation Mount Royal University, Health & Physical Education

Date 11-Jul-2025

COI

Thank you for your revisions. I believe you will agree that it has strengthened your manuscript.
